# Exploring the rules of related parameters in acupuncture for post-stroke dysphagia based on data mining

**DOI:** 10.3389/fneur.2024.1394348

**Published:** 2024-05-24

**Authors:** Minmin Wu, Wenjing Song, Xue Wang, Qiang Tang, Weibin Gao, Luwen Zhu

**Affiliations:** ^1^Department of Rehabilitation Medicine, Heilongjiang University of Chinese Medicine, Harbin, China; ^2^The Second Affiliated Hospital of Heilongjiang University of Chinese Medicine, Harbin, China

**Keywords:** acupuncture, stroke, dysphagia, parameter, data mining RN23, EX-HN14, Gongxue, MS6

## Abstract

**Background:**

Post-stroke dysphagia (PSD) affects the efficacy and safety of swallowing, causing serious complications. Acupuncture is a promising and cost-effective treatment for PSD; however, as the number of randomized controlled trials increases, scientific analysis of the parameters and acupoint prescription is required. Therefore, this study aimed to explore the effects of acupuncture on parameters related to post-stroke dysphagia (PSD).

**Methods:**

We searched the Cochrane Library, PubMed, Embase, Web of Science, China National Knowledge Infrastructure, Wanfang Database, Chinese Biomedical Literature, and Chongqing VIP Database for randomized controlled trials of acupuncture for PSD in the last 15 years and relevant parameters were analyzed using data mining techniques.

**Results:**

In total, 3,205 records were identified, of which 3,507 patients with PSD were included in 39 studies. The comprehensive analysis demonstrated that the closest parameter combinations of acupuncture on PSD were 0.25 mm × 40 mm needle size, 30 min retention time, five treatments per week, and a 4-week total course of treatment. Additionally, the gallbladder and nontraditional meridians, crossing points, and head and neck sites are the most commonly used acupoint parameters. The core acupoints identified were GB20, RN23, EX-HN14, Gongxue, MS6, SJ17, EX-HN12, EX-HN13, and the commonly used combination of EX-HN12, EX-HN13, GB20, and RN23.

**Conclusion:**

This study analyzed the patterns of PSD-related needling and acupoint parameters to provide evidence-based guidelines for clinical acupuncturists in treating PSD, potentially benefitting affected patients.

## 1 Introduction

Dysphagia is a common complication of stroke, and the incidence of post-stroke dysphagia (PSD) ranges from 37–78% (1). PSD impairs the efficacy and safety of swallowing, causing a significant psychological burden and serious complications, such as dehydration, malnutrition, aspiration pneumonia, readmission, and even death (2). In addition to complications and reduced quality of life, PSD increases healthcare costs. A systematic review reported the costs of $16,900 for PSD and $27,600 for pneumonia (3). A prospective cohort study demonstrated that 32.3% of patients with ischemic stroke experienced dysphagia after 7 days, and 80.5% had dysphagia at hospital discharge (4). Thus, managing, preventing, and treating PSD to reduce its incidence, complications, and healthcare costs are current challenges.

Currently, clinical practice guidelines recommend dietary, behavioral (acupuncture), nutritional, pharmacological, and neurostimulatory interventions for PSD (5). Pharmacological treatments can only provide moderate symptom control and often lead to adverse reactions. Although nutritional supplements and neuro-dietary modulation hold clinical significance, the absence of effective markers for malnutrition and challenges in assessing nutritional status are major issues (6). Acupuncture is a promising and cost-effective treatment. A high-quality RCT demonstrated that the cost per quality adjusted life year gained from acupuncture was only 4,241 pounds, well below the commonly accepted threshold of 20,000 pounds. Moreover, sensitivity analyses confirmed a greater than 90% probability of cost-effectiveness, highlighting its economic and therapeutic value (7). Previous meta-analyses have shown that acupuncture re-establishes swallowing function and effectively improves quality of life in patients with PSD (8, 9). Compared to non-acupuncture treatments (swallowing training, medication), acupuncture treatment has a higher efficacy rate (RR, 1.33; 95% confidence interval, 1.25 to 1.43) (8). However, no studies have explored the parameters related to acupuncture in PSD, including the details of acupuncture, core acupoints, and potentially effective combinations of acupoints. Additionally, the standards for reporting interventions in clinical trials of acupuncture (STRICTA) guideline recommend the detailed reporting of the acupuncture parameters implementation process to improve the evaluation of acupuncture efficacy and promote the replication and dissemination of acupuncture studies (10). Therefore, as the number of acupuncture-related randomized controlled trials (RCTs) expands, scientific analyses of the parameters and acupoint prescriptions related to acupuncture for PSD are required.

This study aimed to comprehensively assess the clinical parameters of acupuncture on PSD by conducting a secondary analysis of the literature. Data mining techniques (descriptive analysis, association rules, and cluster analysis) were used to explore the effects of acupuncture on PSD-related parameters, develop standard and effective prescriptions for PSD, reduce confounding factors in the study, and improve the efficacy of acupuncture treatment and the quality of clinical studies.

## 2 Methods

### 2.1 Criteria for considering reviews for inclusion

This systematic review aimed to explore the effects of acupuncture on parameters related to PSD. RCTs on acupuncture interventions for patients with acute or recovering PSD without language restrictions were included. To maintain the rigor of this study, pseudo-randomization, quasi-randomization, experimental studies, reviews, case reports, letters, studies with incomplete data, and publications with duplicate data (data extracted from the most recent literature) were excluded. Studies that did not report any of the parameters of interest were excluded.

All qualifying studies included a control group as a minimum requirement. Furthermore, patients with PSD who underwent acupuncture in the included studies had positive outcomes compared to controls.

### 2.2 Types of participants

PSD is the most frequent condition in which stroke damages the swallowing network, leading to dysphagia (5). Patients with acute (within 7 days) and recovery (within 6 months) PSD were included. Patients with functional dysphagia and dysphagia due to esophageal cancer, laryngeal cancer, or inflammation were excluded. No restrictions based on the type of stroke (hemorrhage or ischemia), location of the lesion (brainstem or non-brainstem), side of the lesion (unilateral or bilateral), age, sex, or geographic region imposed.

### 2.3 Types of interventions and comparisons

Acupuncture therapies in the intervention group included manual acupuncture, electroacupuncture, and conventional physical therapy techniques, whereas those in the control group included rehabilitation, sham acupuncture, placebo acupuncture, or drugs. Studies using moxibustion, acupoint burrowing, auricular acupuncture, and non-invasive interventions (Chinese herbs and tuina) were excluded. No clinical trials comparing different acupuncture methods or acupoint-taking protocols were considered.

### 2.4 Types of outcome measures

Study inclusion in the analysis depended on providing at least one of the following standardized and validated dysphagia scales: the Penetration Aspiration Scale, Functional Oral Intake Scale, Water Swallow Test, Standardized Swallowing Assessment, Videofluoroscopic Swallowing Study, Modified Mann Assessment of Swallowing Ability, Dysphagia Outcome Severity Scale and Swallowing-Quality of Life. Studies that reported only physical or chemical examinations were excluded. The analysis time point was set at the end of all scheduled treatment sessions.

### 2.5 Information sources and search

All articles describing acupuncture and PSD were obtained from the Cochrane Library, PubMed, Embase, Web of Science, China National Knowledge Infrastructure, Wanfang Database, Chinese Biomedical Literature, and Chongqing VIP Database between December 2009 and December 2023 in English and Chinese.

The PubMed and Web of Science search strategies according to population, intervention, control, and outcomes (PICO) format are presented in Table 1. For the other electronic databases, the search approach was adjusted as required.

**Table 1:** Search strategy according to a focused question (PICOS).

Database	Search equation
PubMed	(“Acupuncture” [Mesh] OR “Electroacupuncture” OR “Manual acupuncture” OR “Hand acupuncture”) AND (“Stroke” [Mesh] OR “Cerebral ischemia” OR “Cerebrovascular disease”) AND (“Deglutition Disorders” [Mesh] OR “Dysphagia” OR “Swallowing Disorder”) AND (“Randomized controlled trial” OR “Randomized”)
Web of Science	#1 TS = (Acupuncture) OR AB = (Electroacupuncture) OR AB = (Manual acupuncture) OR AB = (Hand acupuncture) #2 TS = (Stroke) OR AB = (Cerebral ischemia) OR AB = (Cerebrovascular disease) #3 TS = (Deglutition Disorders) OR AB = (Dysphagia) OR AB = (Swallowing Disorder) #4 TS = (Randomized controlled trial) OR AB = (Randomized) #5 #1 AND #2 AND #3 AND #4

### 2.6 Data extraction and selection

After eliminating duplicates, two independent reviewers (MW and WS) screened the literature against the inclusion eligibility criteria (checking the title, abstract, and full text of the papers to identify eligible trials) and cross-checked the screening results. In cases of disagreement, a third reviewer (XW) made the final decision on whether the study should be included.

Data for further analysis were extracted from the included studies: demographic information (title, authors, and year), sample characteristics (age, sex, and patient volume), interventions, and acupuncture parameters (needle type, frequency of treatment, duration of session, duration of stimulation, and acupuncture points).

To evaluate the data on an intention-to-treat basis, missing data were contacted through the corresponding author’s e-mail addresses. If missing data could not be obtained, the currently available literature was excluded.

### 2.7 Literature quality assessment

Two independent reviewers (MW and WS) autonomously evaluated potential biases using “yes,” “no,” or “unclear,” including selection, performance, detection, attrition, reporting, and other biases.

### 2.8 Data synthesis and analysis

#### 2.8.1 Descriptive statistics

Microsoft Excel 2023 was used to conduct statistical descriptive analyses of the relevant parameters in the included studies, including the type of needling, duration of single stimulation, weekly treatment frequency, total treatment duration, and acupoint frequency analysis (acupoint usage, meridian usage, specific acupoint usage, and acupoint location distribution).

#### 2.8.2 Association rule analysis

In this study, the 15 most frequently used acupoints were analyzed. Apriori association rule analysis was conducted using the “arules” and “arulesViz” packages in R software (version 4.3.0). The strength of the association rules met the following criteria: minimum support threshold of 20% and minimum confidence threshold of 90%. Furthermore, if the uplift factor was greater than one, the effect was considered stable.

#### 2.8.3 Complex network analysis

The acupuncture prescriptions were transformed into a vector format, and the vectors were entered into a prescription table in a 0/1 format. These standardized vectors were then imported into SPSS Modeler 18.0, resulting in an association rule stream that included acupoint nodes and their weights. Subsequently, this association rule stream was loaded into Cytoscape 3.10.1 to generate a visual association network of effective acupoints for PSD treatment. Thicker connection lines represent more frequently used acupoints.

#### 2.8.4 Clustering and correlation analysis

R software (version 4.3.0) was used for the clustering and correlation analyses of the 20 acupoints with the highest frequency. Clustering and correlation heatmap analyses were based on the Phi correlation coefficient, which measures the strength and direction of the relationships between binary variables.

## 3 Results

### 3.1 Study selection and characteristics

**Figure 1:**
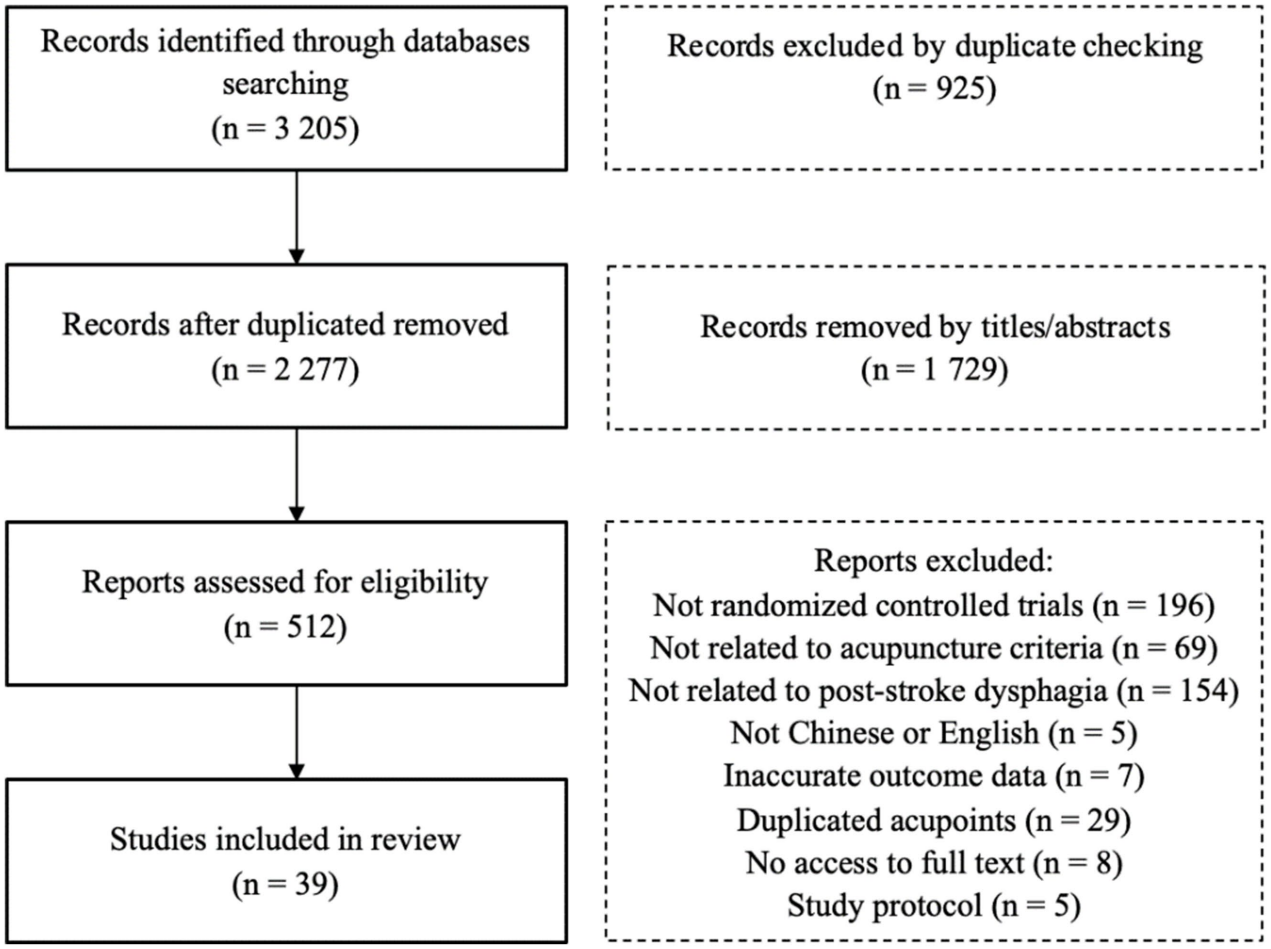
Flowchart of the study identification and selection process.

In total, 3,205 relevant studies were identified. Of these, 925 were duplicates, and 1729 were excluded after screening the titles and abstracts. A total of 512 articles were excluded after full-text evaluation, and 39 articles (11–49) were included for further analysis. The study screening process is visualized using a flowchart (Figure 1).

Detailed characteristics of the included studies are presented in Table 2. Only 3 of the 39 studies were published in English (13, 31, 41), while the rest were published in Chinese. All of these studies were conducted in China over the last 15 years. A total of 3,507 patients with PSD were included, with sample sizes ranging from 21–125 patients. In the treatment group, 27 studies (12–21, 23–25, 27, 28, 30, 31, 33, 35–37, 39, 40, 42, 44, 45, 49) used acupuncture combined with rehabilitation, and seven studies used acupuncture alone (26, 34, 38, 41, 46–48). For the control group, 32 studies used rehabilitation (12–21, 23–25, 27, 28, 30, 31, 33–37, 39–42, 44–49), whereas the remaining studies used sham acupuncture, medication, repetitive transcranial magnetic stimulation, and general nursing care. Regarding numerous outcome indicators, 13 and 27 studies reported the standardized swallowing assessment (SSA) (15–17, 20, 22, 23, 26–28, 31, 40, 46, 47) and water swallow test (WST) (11, 12, 16, 18–21, 23–25, 28–39, 41–43, 45, 47, 49) as valid outcomes, respectively. Nine studies (15, 19, 20, 24, 31, 36, 40, 42, 46) reported swallowing-related quality of life (SWAL-QOL) as an outcome indicator in patients with PSD.

**Table 2:** Characteristics included trials investigating acupuncture in patients with post-stroke dysphagia.

Author (year)	Age, mean, y (I/C)	Sample size (I/C)	Intervention group	Control group	Frequency of retention time (min)	Frequency of treatment (per week)	Frequency of total treatment duration (week)	Outcomes of interest
Chen et al. (13)	62.52/64.06	125/125	MA + Re	Re	30	6 days	3	VFSS, FMA, NIHSS
Xia et al. (40)	65.3/66.1	62/62	MA + Re	Re	30	6 days	4	SSA, DOSS(VFSS), MBI, SWAL-QOL
Liu et al. (31)	67.0/67.1	48/49	MA + Re	Re	30	5 days	8	WST, SSA, SWAL-QOL, RSST
Ren et al. (33)	63.72/64.10	45/45	MA + rTMS	rTMS	20	6 days	4	FOIS, NIHSS, FMA, Barthel Index
Zhang et al. (46)	61.1/60.7	36/36	MA	Re	30	6 days	3	SSA, SWAL-QOL, VFSS
Yu et al. (42)	71/71	21/21	MA + Re + NMES	Re + NMES	30	5 days	3	VDS, WST, FOIS, SWAL-QOL
Huang et al. (23)	NA	50/50	MA + Re	Re	NA	5 days	3	WST, SSA
Huang and Song (22)	57/57	49/49	MA + Me	Me	40	6 days	4	SSA, FDA
Huang and Song (22)	58/57	49/49	MA + Me	Me	40	6 days	4	SSA, FDA
Wang et al. (36)	59.62/60.33	40/40	EA + Re	Re	30	5 days	4	WST, MMASA, SWAL-QOL, sEMG
Chen et al. (15)	62.0/63.0	50/47	MA + Re	Re	30	5 days	4	SSA, SWAL-QOL
Li et al. (28)	61.9/63.6	40/40	MA + Re	Re	30	6 days	4	PAS(FEES), WST, SSA
Wang et al. (37)	65.32/64.76	30/30	MA + Re	Re	30	5 days	4	WST
Fang (18)	58/58	30/30	MA + Re	Re	30	6 days	4	WST
Tong et al. (35)	63/62	30/30	MA + Re	Re	30	5 days	6	WST
Yu et al. (43)	54.30/55.50	30/30	MA + GN	GN	20	5 days	4	WST
Wu et al. (39)	66/68	75/73	MA + Re	Re	30	5 days	4	WST
Li et al. (25)	NA	30/30	MA + Re	Re	30	NA	4	WST
Liu et al. (30)	58.65/58.65	100/100	MA + Re	Re	10	NA	6	WST
Chen and Hu (16)	61.80/ 60.09	35/35	MA + Re	Re	30	5 days	8	WST, SSA, FOIS
Dai et al. (17)	60.47/ 60.74	60/60	MA + Re	Re	10	7 days	8	SSA, NIHSS
Gou and Wang (19)	58.96/ 59.15	44/44	MA + Re	Re	20	7 days	4	WST, FDS, SWAL-QOL
Yuan et al. (44)	62.2/59.9	11/14	MA + Re	Re	30	5 days	2	SSS, MMASA
Zheng and Sun (47)	62.57/61.26	45/45	MA	Re	NA	6 days	4	WST, VFSS, SSA
Zhu et al. (49)	63/64	40/40	MA + Re	Re	30	5 days	4	WST, VFSS
Su and Li (34)	63.77/68.80	30/30	MA	Re	30	6 days	4	WST, FDS, MBI
Bai (12)	63.34/63.15	40/40	MA + Re	Re	20	7 days	4	WST
Li and Yu (27)	58/59	30/30	MA + Re	Re	30	6 days	4	SSA
Zhou et al. (48)	65/64	30/30	MA	Re	30	5 days	2	VFSS
Yu and Hu (45)	63/64	40/38	MA + Re	Re	20	7 days	3	WST
Li et al. (29)	59/62	30/30	MA + GN	GN	NA	7 days	2	WST
Pei (32)	62.12/61.94	39/39	MA + GN	GN	NA	7 days	2	WST
Li et al. (26)	66.4/ 66.6	30/30	MA + Re	Sham MA + Re	30	5 days	4	PAS, SSA, VFSS,
Ji et al. (24)	64.25/63.71	32/32	MA + Re	Re	NA	7 days	2	WST, FDS, SWAL-QOL
He (21)	58.6/ 59.3	33/33	MA + Re	Re	30	7 days	4	WST
Han and Gao (20)	62/63	30/30	MA + Re	Re	30	7 days	2	SSA, WST, SWAL-QOL
Chen and Ni (14)	76.58/ 73.90	40/40	MA + Re	Re	30	7 days	3	RBHOMS, FPBVS
Bahetibek et al. (11)	63.96 /63.87	65/65	MA + GN	GN	5	NA	8	WST
Wei et al. (38)	58.93 /58.25	39/40	MA	Sham MA	NA	6 days	4	WST
Xie et al. (41)	66.0/68.4	70/70	MA	Re	30	5 days	4	WST

MA, Manual Acupuncture; Re, Rehabilitation; rTMS, repetitive Transcranial Magnetic Stimulation; Me, Medicine; EA, Electropuncture; GN, General Nursing; WST, Water Swallow Test; SSA, Standardized Swallowing Assessment; SWAL-QOL, Swallowing-Related Quality of Life; VFSS, Video-fluoroscopic Swallowing Study; DOSS, Dysphagia Outcome Severity Scale; MBI, Modified Barthel Index; FOIS, Functional Oral Intake Scale; FEES, Fiberoptic Endoscopic Evaluation of Swallowing; PAS, Penetration Aspiration Scale; SSS, Swallow Severity Scale; FDS, Fujishima Dysphagia Scale; RBHOMS, Royal Brisbane Hospital Outcome Measure for Swallowing; FPBVS, Food Properties and Beverage Viscosity Scores; FMA, Fugl-Meyer Assessment; NIHSS, National Institutes of Health Stroke Scale; FDA, Frenchay Dysarthria Assessment-2; RSST, Repetitive Saliva-Swallowing Test; sEMG, Surface Electromyography; MMASA, Modified Mann Assessment of Swallowing Ability; NMES, Neuromuscular Electrical Stimulation; VDS, Videofluoroscopic Dysphagia Scale; NA, Not Available.

All 39 included RCTs reported positive outcomes, supporting the therapeutic effectiveness of acupuncture for patients with PSD.

### 3.2 Risk of bias assessment

**Figure 2:**
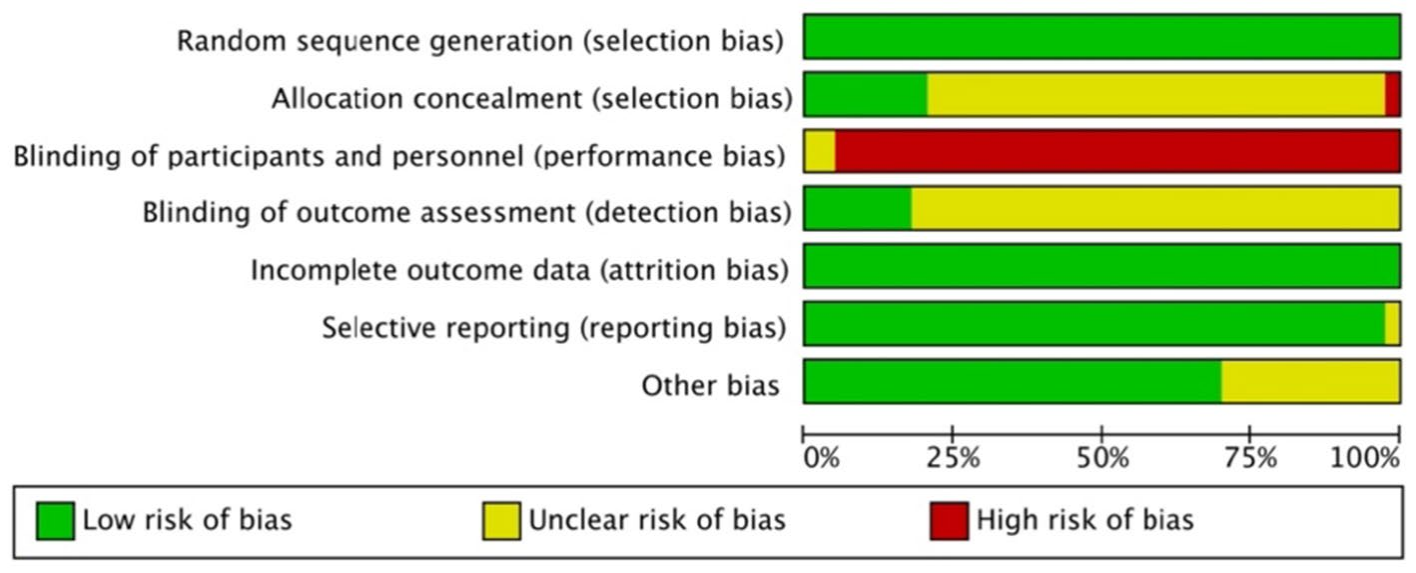
Risk of bias assessment.

All studies reported randomized allocation methods and no pseudo- or quasi-RCTs. Eight studies reported allocation concealment (13, 15, 31, 35, 39–42), except for one, which had a high risk of bias (46), whereas the remaining studies did not report allocation concealment, giving an unclear risk of bias. The acupuncturists were almost impossible to blind, and the remaining literature had a high risk of bias, except for two English-language papers, which were unclear about the risk of bias (13, 31). Seven studies reported a detection bias (13, 14, 31, 40–42, 46), and the remaining studies reported an unclear risk of bias. In the incomplete outcome and selective reporting section, the remaining studies gave a low risk of bias, except for one that gave unclear selective reporting (41). Regarding other biases, 12 studies reported an unclear risk of bias (11, 12, 24, 27, 29, 30, 35, 38, 43–45, 47), while the remaining studies reported a low risk of bias (Figure 2).

### 3.3 Frequency of needle type

Eleven needle types were analyzed in 39 studies with a frequency of 27 uses. However, 21 studies did not report the needle type, which may have affected the RCT replication. Notably, one study reported needle-type unavailability, which was 0.20–0.25 mm × 25–75 mm. The most used needle type in these studies was 0.25 mm × 40 mm with a frequency of eight uses, accounting for 30% of the total frequency. Six needle types were used at a frequency of 1 and 6 for almost more than half of the needle types (Table 3).

**Table 3:** Frequency of needle type.

No.	Needle type	Frequency	Percentage
1	0.25 mm × 40 mm	8	0.30
2	0.30 mm × 40 mm	5	0.19
3	0.25 mm × 25 mm	4	0.15
4	0.25 mm × 50 mm	2	0.07
5	0.35 mm × 75 mm	2	0.07
6	0.20 mm × 25 mm	1	0.04
7	0.30 mm × 50 mm	1	0.04
8	0.30 mm × 70 mm	1	0.04
9	0.30 mm × 75 mm	1	0.04
10	0.40 mm × 50 mm	1	0.04
11	0.40 mm × 60 mm	1	0.04

### 3.4 Retention time

Five retention times were observed in 39 studies, with the longest being 30 min and the shortest being 5 min. The 30 min retention time was used as frequently as 24 times or 71%. In clinical practice, the choice of 30 min of acupuncture intervention for PSD may have the intended effect. Although only 5 min occurred once, the study yielded positive results. Notably, retention time was not available in six studies (Table 4).

**Table 4:** Frequency of retention time.

No.	Retention time (min)	Frequency	Percentage
1	30	24	0.71
2	20	5	0.15
3	10	2	0.06
4	40	2	0.06
5	5	1	0.03

### 3.5 Frequency of treatment

The results of the frequency analysis of treatments in the 39 studies showed four types, which were 5, 6, 7, and 3 days per week. The highest frequency was 5 days per week, and the lowest frequency was 3 days per week. Except for 3 days per week, the frequency share of the other treatments was approximately 0.3. However, the frequency of treatment was not available in 3 out of 39 studies (Table 5).

**Table 5:** Frequency of treatment.

No.	Frequency of treatment	Frequency	Percentage
1	5 days per week	15	0.39
2	6 days per week	12	0.32
3	7 days per week	10	0.26
4	3 days per week	1	0.03

### 3.6 Total treatment duration

A total of 39 studies included five acupuncture interventions for PSD, all of which reported the total treatment time. Four weeks of intervention were used as frequently as 22 times, which was more than half of all frequencies. Six and 8 weeks total intervention time frequency of 6 times is nearly 20%. This may indicate that the PSD recovered better and for a shorter duration with acupuncture interventions (Table 6).

**Table 6:** Frequency of total treatment duration.

No.	Total treatment duration	Frequency	Percentage
1	3 weeks	22	0.55
2	2 weeks	6	0.15
3	3 weeks	6	0.15
4	8 weeks	4	0.10
5	6 weeks	2	0.05

### 3.7 Acupoint distribution

**Figure 3:**
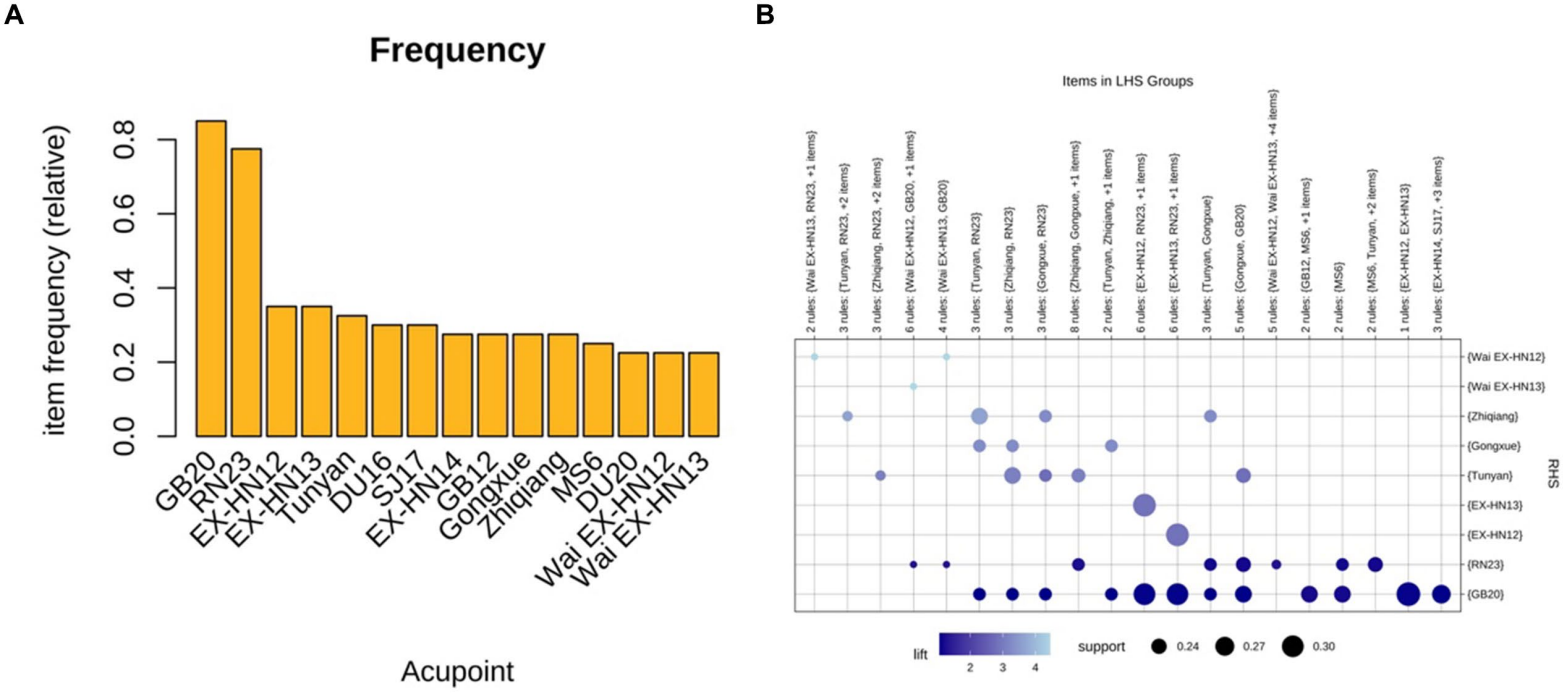
**(A)** Frequency of the top 15 acupoints used for PSD. **(B)** Grouping matrix of 72 association rules for acupuncture intervention PSD.

Forty PSD-eligible intervention prescriptions, 71 acupoints, and 322 occurrences were identified in 39 studies. The top 15 acupoints included Fengchi (GB20), Lianquan (RN23), Jinjin (EX-HN12), Yuye (EX-HN13), Tunyan (new acupoint), Yifeng (SJ17), Yiming (EX-HN14), Wangu (GB12), Gongxue (new acupoint), Zhiqiang (new acupoint), Fengfu (DU16), Anterior oblique line of vertex-tempora (MS6), Baihui (DU20), Wai jinjin (Wai EX-HN12), and Wai yuye (Wai EX-HN13) (Table 7 and Figure 3A).

**Table 7:** Frequency of the top 15 acupoints used for PSD.

No.	Acupoint	Frequency	Meridians	Site of the point	Specific points
1	GB20	34	GB	Head, face, and neck	Crossing point
2	RN23	31	RN	Head, face, and neck	Crossing point
3	EX-HN12	14	EX	Head, face, and neck	NA
4	EX-HN13	14	EX	Head, face, and neck	NA
5	Tunyan	13	Other	Head, face, and neck	NA
6	SJ17	12	SJ	Head, face, and neck	Crossing point
7	EX-HN14	11	EX	Head, face, and neck	NA
8	GB12	11	GB	Head, face, and neck	NA
9	Gongxue	11	Other	Head, face, and neck	NA
10	Zhiqiang	11	Other	Head, face, and neck	NA
11	DU16	12	DU	Head, face, and neck	Crossing point
12	MS6	10	MS	Head, face, and neck	NA
13	DU20	9	DU	Head, face, and neck	Crossing point
14	Wai EX-HN12	9	Other	Head, face, and neck	NA
15	Wai EX-HN13	9	Other	Head, face, and neck	NA

Of the 40 acupoint prescriptions, 46 were used by 14 conventional meridians. Specifically, there were 12 conventional meridians, 1 Conception Vessel (RN), 1 Governor Vessel (DU), and nonconventional meridians. Notably, nonconventional meridian acupoints accounted for the highest number of acupoints (19) and were used as frequently as 80 times. Although not conventional, the high frequency of new acupoints indicates that clinical experience should not be ignored. In the meridian analysis, LU, LR, and HT meridians were all used twice, and all were one acupoint. Although GB had a frequency of 45 usages, only two acupoints (GB20 and GB12) may be potentially valuable for acupuncture interventions in PSD. Moreover, the SJ, ST, LI, BL, PC, SP, KI, LU, LR, and HT meridians were used <5% of the total frequency, and these meridians may be less appropriate for acupuncture intervention in PSD (Table 8).

**Table 8:** Frequency and percentage of meridians used for PSD.

Meridians	Frequency	Percentage	Acupoint number	Acupoints (frequency)
Other	80	0.25	19	Tunyan (13), Gongxue (11), Zhiqiang (11), Wai EX-HN12 (9), Wai EX-HN13 (9), Pang RN23 (5), Fayin (4), Shang RN23 (4), Yanhoubi (3), Tiyan (2), Aqiang (1), Ganqu (1), Piqu (1), Shangjiaoqu (1), Shezhong (1), Siqiang (1), Tunyan2 (1), Xinqu (1), Zhifanliu (1)
GB	45	0.14	2	GB20 (34), GB12 (11)
EX	43	0.13	6	EX-HN12 (14), EX-HN13 (14), EX-HN14 (11), EX-B2 (2), EX-HN11 (1), EX-HN5 (1)
RN	43	0.13	6	RN23 (31), RN22 (7), RN24 (2), RN12 (1), RN4 (1), RN6 (1)
DU	31	0.10	7	DU16 (12), DU20 (9), DU26 (4), DU15 (3), DU17 (1), DU24 (1), DU29 (1)
MS	20	0.06	5	MS6 (10), MS7 (6), MS5 (2), MS1 (1), MS9 (1)
SJ	13	0.04	2	SJ17 (12), SJ5 (1)
ST	12	0.04	6	ST9 (5), ST36 (2), ST40 (2), ST25 (1), ST4 (1), ST6 (1)
LI	9	0.03	4	LI4 (6), LI11 (1), LI14 (1), LI18 (1)
BL	6	0.02	5	BL10 (2), BL13 (1), BL18 (1), BL20 (1), BL23 (1)
PC	5	0.02	1	PC6 (5)
SP	5	0.02	2	SP6 (4), SP4 (1)
KI	4	0.01	3	KI6 (2), KI1 (1), KI3 (1)
LU	2	0.01	1	LU7 (2)
LR	2	0.01	1	LR3 (2)
HT	2	0.01	1	HT5 (2)

GB, Gallbladder Meridian of Foot Shaoyang; EX, Extra points Meridian; RN, Conception Vessel; DU, Governor Vessel; MS, Anterior parietotemporal oblique line, SJ, San Jiao Meridian of Hand Shaoyang; ST, Stomach Meridian of Foot Yangming; LI, Large Intestine Meridian of Hand Yangming; BL, the Bladder Meridian of Foot Taiyang; PC, Pericardium Meridian of Hand Jueyin; SP, Spleen Meridian of Foot Taiyin; KI, Kidney Meridian of Foot Shaoyin; LU, Lung Meridian of Hand Taiyin; LR, Liver Meridian of Foot Jueyin; HT, Heart Meridian of Hand Shaoyin.

Of the 71 acupoints, 44 were specific, and 10 contained more than one attribute. Specifically, PC6, LU7, SJ5, and SP4 were Luo-connecting points and eight confluent points. RN12 possesses the attributes of eight influential points: the front Mu and crossing points. The frequency, percentage, and highest number of acupoints used as crossing points in the specific-point analysis were 123, 38%, and 15, respectively. Acupuncturists should combine the crossing points in clinical practice to achieve better therapeutic outcomes. The Yuan primary point and Luo connecting point also had a moderate frequency in this study: 13 and 9 times, respectively. Notably, the frequencies of the eight influential points, lower He-Sea points, front Mu points, and back Shu points were <0.01, indicating that clinical acupuncturists seldom considered these specific acupoints for therapeutic PSD (Table 9).

**Table 9:** Frequency and percentage of specific acupoints used for PSD.

Specific acupoints	Frequency	Percentage	Acupoint number	Acupoints (frequency)
Crossing point	123	0.38	15	GB20 (34), RN23 (31), SJ17 (12), DU16 (12), DU20 (9), RN22 (7), DU26 (4), SP6 (4), DU15 (3), RN24 (2), DU17 (1), DU24 (1), RN12 (1), RN4 (1), ST4 (1)
Luo-connecting point	13	0.04	6	PC6 (5), HT5 (2), LU7 (2), ST40 (2), SJ5 (1), SP4 (1)
Eight confluent point	11	0.03	5	PC6 (5), KI6 (2), LU7 (2), SJ5 (1), SP4 (1)
Yuan-primary point	9	0.03	3	LI4 (6), LR3 (2), KI3 (1)
Five Shu points	7	0.02	5	LR3 (2), ST36 (2), KI1 (1), KI3 (1), LI11 (1)
Back Shu point	4	0.01	4	BL13 (1), BL18 (1), BL20 (1), BL23 (1)
Front Mu point	3	0.01	3	RN12 (1), RN4 (1), ST25 (1)
Lower He-Sea point	3	0.01	2	ST36 (2), LI11 (1)
Eight influential point	1	0.00	1	RN12 (1)

In the acupoints distribution analysis, the head, face, and neck were the most selected areas, with frequencies, percentages, and acupoints of 279, 87%, and 47, respectively. This area is consistent with the clinical symptoms of patients with PSD, indicating that acupuncturists tend to consistently select the acupoint locations. The chest, abdomen, and back had very few acupoints, nine in total, and these locations were not considered, possibly because of disease specificity (Table 10).

**Table 10:** Frequency and percentage of acupoint sites used for PSD.

Site of acupoints	Frequency	Percentage	Acupoint number	Acupoints (frequency)
Head, face, and neck	279	0.87	47	GB20 (34), RN23 (31), EX-HN12 (14), EX-HN13 (14), Tunyan (13), SJ17 (12), DU16 (12), EX-HN14 (11), GB12 (11), Gongxue (11), Zhiqiang (11), MS6 (10), DU20 (9), Wai EX-HN12 (9), Wai EX-HN13 (9), RN22 (7), MS7 (6), Pang RN23 (5), ST9 (5), DU26 (4), Fayin (4), Shang RN23 (4), DU15 (3), Yanhoubi (3), EX-B2 (2), MS5 (2), RN24 (2), Tiyan (2), Aqiang (1), DU17 (1), DU24 (1), DU29 (1), EX-HN10 (1), EX-HN5 (1), Ganqu (1), LI18 (1), MS1 (1), MS9 (1), Piqu (1), Shangjiaoqu (1), Shezhong (1), Siqiang (1), ST4 (1), ST6 (1), Tunyan2 (1), Xinqu (1), Zhifanliu (1)
Upper limbs	18	0.06	7	LI4 (6), PC6 (5), HT5 (2), LU7 (2), LI11 (1), LI14 (1), SJ5 (1)
Lower limbs	15	0.05	8	SP6 (4), KI6 (2), LR3 (2), ST36 (2), ST40 (2), KI1 (1), KI3 (1), SP4 (1)
Back	6	0.02	5	BL10 (2), BL13 (1), BL18 (1), BL20 (1), BL23 (1)
Chest and abdomen	4	0.01	4	RN12 (1), RN4 (1), RN6 (1), ST25 (1)

### 3.8 Association rule mining analysis

Association rule analysis of the Apriori algorithm was performed using R software (version 4.3.0) to obtain 72 association rules. A grouping matrix plot was used to visualize the 72 association rules (Figure 3B). Darker purple circles indicate higher degrees of lift, whereas larger circles indicate higher support.

Based on the minimum support threshold of 20%, the minimum confidence threshold of 90%, and an uplift factor of>1, 20 pairs of acupoint combinations with the highest support thresholds in the PSD prescriptions were summarized. The first 12 pairs included {EX-HN12} ≧ {EX-HN13}, {EX-HN13} ≧ {EX-HN12}, {EX-HN12} ≧ {GB20}, {EX-HN13} ≧ {GB20}, {EX-HN12, EX-HN13} ≧ {GB20}, {EX-HN12, GB20} ≧ {EX- HN13}, {EX-HN13, GB20} ≧ {EX-HN12}, {EX-HN12, RN23} ≧ {EX-HN13}, {EX-HN13, RN23} ≧ {EX-HN12}, {GB12} ≧ {GB20}, {Zhiqiang} ≧ {Tunyan}, and {SJ17} ≧ {GB20}. The top 20 pairs of acupoint combinations had confidence, support, and lift values (Table 11), and more than half of the acupoint combinations had confidence values of 100%. Notably, all 20 pairs of acupoint combinations had lift values >1, with nine acupoint combinations >2.

**Table 11:** The top 20 acupoint association rules for PSD treatments.

No.	Association rules	Support	Confidence	Lift
1	{EX-HN12} ≧ {EX-HN13}	0.35	1.00	2.86
2	{EX-HN13} ≧ {EX-HN12}	0.35	1.00	2.86
3	{EX-HN12} ≧ {GB20}	0.33	0.93	1.09
4	{EX-HN13} ≧ {GB20}	0.33	0.93	1.09
5	{EX-HN12, EX-HN13} ≧ {GB20}	0.33	0.93	1.09
6	{EX-HN12, GB20} ≧ {EX-HN13}	0.33	1.00	2.86
7	{EX-HN13, GB20} ≧ {EX-HN12}	0.33	1.00	2.86
8	{EX-HN12, RN23} ≧ {EX-HN13}	0.30	1.00	2.86
9	{EX-HN13, RN23} ≧ {EX-HN12}	0.30	1.00	2.86
10	{GB12} ≧ {GB20}	0.28	1.00	1.18
11	{Zhiqiang} ≧ {Tunyan}	0.28	1.00	3.08
12	{SJ17} ≧ {GB20}	0.28	0.92	1.08
13	{EX-HN12, RN23} ≧ {GB20}	0.28	0.92	1.08
14	{EX-HN13, RN23} ≧ {GB20}	0.28	0.92	1.08
15	{EX-HN12, EX-HN13, RN23} ≧ {GB20}	0.28	0.92	1.08
16	{EX-HN12, GB20, RN23} ≧ {EX-HN13}	0.28	1.00	2.86
17	{EX-HN13, GB20, RN23} ≧ {EX-HN12}	0.28	1.00	2.86
18	{MS6} ≧ {GB20}	0.25	1.00	1.18
19	{EX-HN14} ≧ {GB20}	0.25	0.91	1.07
20	{Zhiqiang} ≧ {RN23}	0.25	0.91	1.17

### 3.9 Complex network analysis

**Figure 4:**
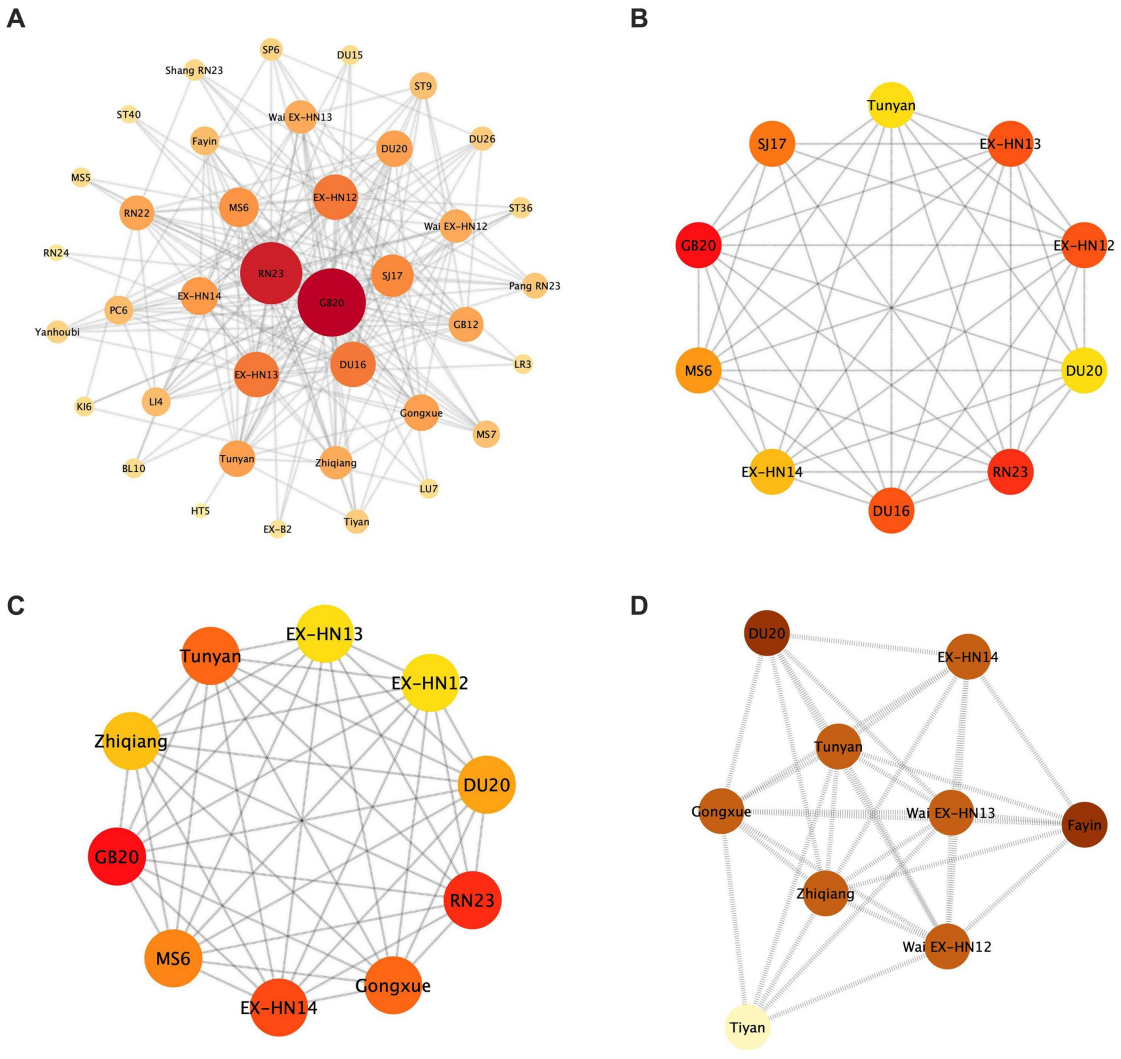
Complex network analysis. **(A)** Complex network analysis of acupuncture for PSD treatment. **(B)** MNC shows the top 10 acupuncture points. **(C)** MCC shows the top 10 acupuncture points. **(D)** MCODE shows 9 acupoints and 32 edges.

A complex network analysis was performed using Cytoscape to derive 38 acupoints (nodes) and 210 edges, which were classified into four layers by degree scores: the darker the color and the higher the frequency. Hub acupoints were obtained using the maximal clique centrality (MCC), maximum neighborhood component (MNC), and molecular complex detection tool (MCODE). Specifically, the top 10 acupoints and 45 edges were obtained using MCC in the Cytoscape tool, with the acupoints in descending order as follows: GB20, RN23, EX-HN12, EX-HN13, Tunyan, EX-HN14, Zhiqiang, Gongxue, MS6, and DU20. The top 10 acupoints and 42 edges were obtained using MNCs, including GB20, RN23, EX-HN12, EX-HN13, Tunyan, SJ17, DU16, EX-HN14, MS6, and DU20. Additionally, nine acupoints and 32 edges were obtained using MCODE, including DU20, Fayin, Tunyan, EX-HN14, Zhiqiang, Wai EX-HN12, Gongxue, Wai EX-HN13, and Tiyan. Notably, these hub acupoints were consistent with the results of association rule analysis (Figure 4).

### 3.10 Correlation and cluster analysis

**Figure 5:**
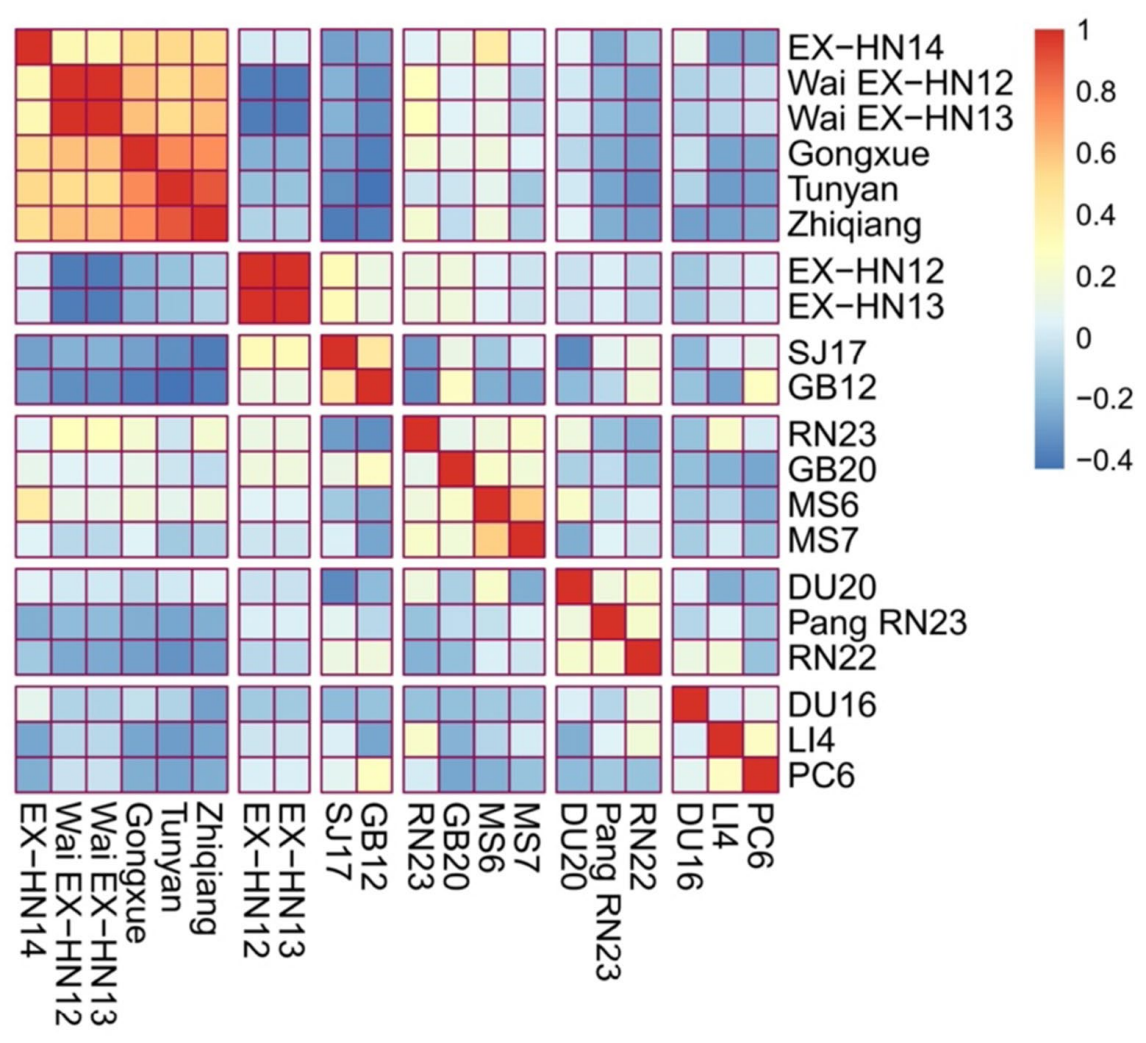
Correlation and cluster analysis of the top 20 frequently selected acupoints.

The 20 most frequently used acupoints were clustered and correlated into six clusters. Cluster 1 includes EX-HN14, Wai EX-HN12, Wai EX-HN13, Gongxue, Tunyan, and Zhiqiang. Cluster 2 comprised EX-HN12 and EX-HN13. Cluster 3 included strains SJ17 and GB12. Cluster 4 includes RN23, GB20, MS6, and MS7. Cluster 5 included DU20, Pang RN23, and RN22. Cluster 6 included DU16, LI4, and PC6 (Figure 5).

## 4 Discussion

We organized and established a database of unstructured data from studies of acupuncture intervention in PSD using data mining techniques to summarize the parameters related to acupuncture in PSD over the past 15 years and to provide evidence-based guidance for future clinical data studies and methodological research. Specifically, we integrated 39 RCTs of acupuncture intervention for PSD and extracted needling parameters (needling type, retention time, treatment frequency, and treatment period) and acupoint parameters (frequency of acupoints, meridians, specific acupoints, and acupoint sites) from the studies with positive outcomes. Moreover, the correlation rules, complex networks, clustering, and correlations of the acupoints were analyzed. These results have positive implications for acupuncturists when making prescription decisions for the treatment of PSD.

Regarding needling parameter patterns, we identified the two most frequently used needling types, 0.25 mm × 40 mm and 0.30 mm × 40 mm, and the optimal retention time of 30 min. Clinical acupuncturists varied somewhat in their choice of needle type and retention time owing to inconsistent practice backgrounds. However, our results may indicate an overall trend in acupuncture parameters for PSD. Although the frequency of treatment suggests that five times per week is optimal, recent studies have demonstrated different after-effects of acupuncture at different intervals (50). Specifically, short intervals (6 h) somewhat blocked acupuncture after the effects, and long intervals (48 h) prolonged them. Regarding the total intervention, a four-week intervention may be optimal for the recovery of patients with PSD. This result may depend on inconsistent point selection criteria by acupuncturists, and the efficacy may be affected by confounding factors.

Regarding acupoint parameters, the commonly used acupoints were GB20 and RN23. Internally, it passes through the branches of the greater occipital nerve, the lesser occipital nerve, and the occipital artery, and it is innervated by the vagus nerve and glossopharyngeal nerve. Acupuncture of the GB20 stimulates the neck muscles, improves blood supply to the vertebrobasilar artery system, increases blood flow to the brain, promotes nerve repair and regeneration, and restores swallowing strength in patients with PSD (51, 52). Anatomically, RN23 is surrounded by the hyoid muscle, genioglossus muscle, glossopharyngeal nerve, and hypoglossal nerves. Acupuncture stimulates local nerve points, rebuilds the reflex arc of the central nervous system excitability, restores pharyngeal innervation, and completes involuntary movement (53, 54). Moreover, the GB, EX, RN, and nontraditional meridians are most frequently used in the clinical treatment of PSD. These meridians pass through the pharynx, and needling acupoints on the meridians reflect the traditional medical principle of “wherever the meridians pass, the main treatment will be applied.” Joint regulation of multiple meridians promotes blood circulation in the glossopharynx and restores normal swallowing function. Compared to the lower He-Sea point, five Shu points, and eight influential points, the crossing point is a more important acupoint in PSD selection. For example, GB20 belongs to the GB meridian, which is the meeting point for the GB, Sanjiao, and Yangwei meridians. Acupuncture of the GB meridian can simultaneously regulate all three meridians, which play a role in the recovery of patients with PSD.

Our findings indicate that the acupoints are mostly located on the head, face, and neck, which is consistent with the etiology of dysphagia (mostly cerebrovascular disease) and the location of lesions (located in the cerebral cortex or subcortex). Thus, needling the head acupoints stimulates the corresponding swallowing function area of the brain, which can improve cerebral blood flow, regulate growth factors, reduce inflammatory factors, and promote the recovery of neuronal cells in the lesion location of the brain (55). Additionally, needling peripheral areas, such as the face and neck, stimulate multiple brain nerves that innervate swallowing movements, such as the trigeminal, facial, glossopharyngeal, vagus, and hypoglossal nerves. These stimulation signals are transmitted to the swallowing center and cerebral cortex, which promote compensatory brain function and recovery of the swallowing muscles. Furthermore, acupoint association rule analysis identified the most strongly associated core acupoints: GB20, RN23, EX-HN14, Gongxue, MS6, SJ17, EX-HN12, and EX-HN13. Data mining analysis using frequency counts, clustering, and correlation indicated that the acupoints with the strongest evidence of acupuncture in the PSD group were GB20, RN23, and MS6. Our study provides more choices of parameters for acupuncture in PSD, and the specificity of acupoints should be emphasized based on the effectiveness obtained by acupuncturists in clinical practice.

Qiao and colleagues measured the characteristics of dysphagia in individuals with stroke at different lesion locations through the duration, movement, and swallowing function using Videofluoroscopic Swallow Study (56). The report indicates that patients with infratentorial strokes had worse swallowing functions compared to those with supratentorial strokes. However, a single-center retrospective study showed that the risk of PSD was reduced in patients receiving acupuncture treatment, regardless of whether the stroke occurred in the brainstem [adjusted hazard ratio (AHR) = 0.41], thalamus (AHR = 0.13), or was a multifocal lesion (AHR = 0.40) (57). Although the results indicate that acupuncture treatment can reduce the risk of dysphagia in stroke patients across various demographics, including different ages, sexes, stroke types, sites, and baseline comorbidities (57), we recognize the need to further elucidate the application of acupuncture techniques in diverse clinical settings. Additionally, our standardized treatment protocols require further evaluation in future research to determine their effectiveness and applicability.

This study had some limitations that should be interpreted with caution by clinicians and researchers. First, 27 studies reported the WST as an outcome indicator; however, the guidelines for PSD suggest that the WST should be used for initial screening. Instrumentation (FESS and VFSS), combined with scale interpretation as an outcome assessment, can objectively confirm the degree of recovery from dysphagia. Second, most studies did not report the practice of acupuncturists, and untrained or inexperienced acupuncturists may result in different therapeutic and physiological outcomes. Future studies should follow the STRICTA guidelines to improve the quality of the evidence. Third, the 39 RCTs included poorer methodological quality, lack of allocation concealment, and inability to confirm preregistered protocols, which would carry some risk of bias. Finally, we performed strong data integration of acupuncture type, treatment duration, and acupoint association. However, further large-sample RCTs or animal experiments are needed to validate our results.

## 5 Conclusion

This study utilized data mining methods to summarize the optimal parameters and clinical acupuncture point selection characteristics for acupuncture treatment of PSD. The closest parameter combination included needle sizes of 0.25 mm × 40 mm, a needle retention time of 30 min, treatment frequency of five times per week, and a total treatment course of four weeks. Additionally, the core points identified were GB20, RN23, EX-HN14, Gongxue, MS6, SJ17, EX-HN12, and EX-HN13, with the principal combinations being EX-HN12, EX-HN13, GB20, and RN23. Due to the limitations of this study, further research and more standardized clinical trials are still needed to guide and optimize acupuncture treatment plans, providing a theoretical basis for clinical acupuncturists.

## Data availability statement

The original contributions presented in the study are included in the article/supplementary material, further inquiries can be directed to the corresponding author.

## Author contributions

MW: Conceptualization, Data curation, Software, Writing – original draft, Writing – review & editing. WS: Formal analysis, Investigation, Methodology, Writing – review & editing. XW: Investigation, Methodology, Visualization, Writing – review & editing. QT: Conceptualization, Supervision, Validation, Writing – review & editing. WG: Supervision, Validation, Writing – review & editing. LZ: Conceptualization, Funding acquisition, Project administration, Supervision, Validation, Writing – review & editing.
